# Supplementary material for: Genetics re-establish the utility of 2-methylhopanes as cyanobacterial biomarkers before 750 million years ago
Source: Nat Ecol Evol. 2023 Oct 26;7(12):2045–54. doi: 10.1038/s41559-023-02223-5 (PMC10697835; doi:10.1038/s41559-023-02223-5)
Supplement: Supplementary file 1 — Supplementary Notes 1−9, Figs. 1−12, References (Supplementary Table 7 references included) and captions for Tables 1–7. [file 41559_2023_2223_MOESM1_ESM.pdf]

nature ecology & evolution

Article https://doi.org/10.1038/s41559-023-02223-5

Genetics re-establish the utility of 2-methylhopanes as cyanobacterial biomarkers before 750 million years ago

In the format provided by the authors and unedited

This PDF file includes:

Supplementary Notes 1−9
Supplementary Information references
Supplementary Figs. 1−12
Supplementary Tables 1−7 captions

Supplementary Note 1: Distribution of SC and HpnP in bacteria

Although squalene cyclase (SC) and HpnP are distributed in 31 and 12 bacterial phyla, respectively, the majority of HpnP-containing phyla contain only a handful of species that possess HpnP and these atypical species likely acquired HpnP via horizontal gene transfer (HGT). Hence, HpnP is essentially concentrated in three phyla: Rokubacteria, Alphaproteobacteria and Cyanobacteria (Fig. 1a). Yet also in these three phyla, the gene is not universally distributed. Currently, no complete genome is available for Rokubacteria, but 117 out of 140 species that have draft genome data were found to possess SC (84%), and HpnP was present in 50 species among those SC-containing species (42%) (Supplementary Table 1). Six rokubacterial species were found to possess only HpnP (Supplementary Table 1), but it is not clear if the apparent lack of SC is due to the incomplete genome sequencing status or alternatively if HpnP in those species have a function that is irrelevant to hopanoid biosynthesis. In Alphaproteobacteria, SC is widespread (but not ubiquitous) in the phylum, while HpnP is mostly constrained to a single order (Hyphomicrobiales; former Rhizobiales) (Fig. 1B)—in particular to three out of 17 families in the order, as was previously observed (Beijerinckiaceae, Bradyrhizobiaceae and Methylobacteriaceae; Supplementary Tables 2 and 3)[1]. The majority of species in these three families contain both SC and HpnP. Hyphomicrobiales represents a late-branching taxon in the phylum[2] and thus a late origin of HpnP in Alphaproteobacteria via HGT is inferred. Among 328 species within the entire Hyphomicrobiales for which complete genome data are available, only 93 species (28%) were found to contain SC; the majority of those SC-containing species (71 species; 76%) harbor both SC and HpnP (Supplementary Table 2). Although the inclusion of draft genome data increases the number of species that contain SC and HpnP, the distribution of HpnP is still mostly limited to the three families described above (Supplementary Table 2).

In Cyanobacteria, HpnP is found in most taxonomic lineages (Fig. 1B), although the distribution of SC and HpnP is not universal even in this phylum. Among 171 species that have complete genome data, 70 species (41%) contain SC (Supplementary Tables 4–5). Approximately half of SC-containing cyanobacteria (32 species; 46%) were found to additionally possess HpnP (Supplementary Tables 4–5). The distribution of SC and HpnP varies between different clades. For instance, the genus Nostoc contains 19 species that have complete genome data. While SC is found in all of 19 species, HpnP is found in 13 species (68%). In contrast, in the genus Leptolyngbya, three out of six species that have complete genome data have both SC and HpnP (50%), while the other three lack both genes. If only complete genomes are considered, only 19 out of 38 cyanobacterial families (50%) harbor SC-containing species and nine families (24%) harbor species that have both SC and HpnP. However, if draft genomes are included, these values rise to 30 and 27 families (79% and 71%), respectively. Hence, the occurrence of SC and in particular HpnP seems to be underestimated, if based only on complete genomes, due to the sporadic distribution of these two genes. The completion of more cyanobacterial genome sequencing would likely further increase the number of HpnP-containing species in Cyanobacteria.

Supplementary Note 2: Triterpenoid-related proteins in myxobacteria

Instead of SC, several species of myxobacteria that have a HpnP homolog have oxidosqualene cyclase (OSC), which is evolutionarily and functionally related to SC and is involved in steroid biosynthesis[3]. However, the distribution of HpnP homologs is punctate in myxobacteria and has no correlations to the distribution of OSC in the lineage. Additionally, no 2-methylated steroids have been observed in myxobacteria thus far. Hence, it is not likely that the HpnP homolog in

myxobacteria is involved in steroid biosynthesis and the function of myxobacterial HpnP homologs remains unknown.

<u>Supplementary Note 3</u>: HpnP gene loss in Cyanobacteria

While it is inferred that HpnP was present in the common ancestor of Cyanobacteria and was vertically inherited for the majority of HpnP-containing species, this does not necessarily mean that HpnP was pervasive in early cyanobacteria. For instance, the distribution of HpnP in stem-group lineages as well as extinct crown-group lineages cannot be estimated since their genetic information is entirely lost. Within crown-group cyanobacteria, HpnP gene loss seems to have occurred many times in multiple lineages. It is currently unknown what driving force caused the gene loss in association with a particular environmental setting.

The role of 2-methylhopanoids is thought to be related to stress response, as observed for the elevated production of 2-methylhopanoids by a plant-associated alphaproteobacterium under hypoxic and acidic conditions[4]. Hence, the loss of HpnP may imply the disappearance of a particular environmental stressor for host organisms. For instance, early aerobic organisms may have experienced more frequent and persistent hypoxic conditions and thus HpnP-induced cell protection may have been more advantageous than in later oxygenated worlds. However, it is not clear if this specific observation for terrestrial species can be applicable to organisms in other habitats, in particular marine species. The apparent low abundance of HpnP in marine species in previous studies[5] might suggest a link of 2-methylhopanoids to low-salinity environments. However, in our study, HpnP is found to be more common in marine SC-containing species than previously thought (Supplementary Note 5 for more details), but instead hopanoid production in general seems to be less common in marine species, relative to terrestrial species. Elucidating the general relationship of (methyl)hopanoid production to environmental stress (*e.g.* oxygen, salinity) would thus be an important topic for future study.

The relationship of HpnP gene loss to the Neoproterozoic ecological shift is another important topic because the dramatic shift in the 2-MHI apparently occurs in parallel with the rise of modern marine planktonic cyanobacteria. In our present study, the dates of a few relevant HpnP gene loss events were estimated, utilizing a recently-published phylogenomic study of marine cyanobacteria (Extended Data Fig. 2)[6]. The lineage that contains marine *Synechococcus*/*Prochlorococcus* species is likely to have initially been associated with freshwater environments[7]. The only HpnP-containing species in the lineage is *Prochlorothrix hollandica PCC 9006*. However, the divergence of this freshwater species from the rest of the lineage is around 1.5 Ga and thus is well before the emergence of the marine planktonic clade in the Neoproterozoic (light blue dot; Extended Data Fig. 2)[6,7]. The phylogeny of Cyanobacteria is not completely settled and thus the dating may not be accurate, but the tree topology for several key sub-clades, including the marine planktonic lineage, is generally well supported[6,8]. Hence, the loss of HpnP was probably not related to the evolution of the marine planktonic clade. Yet, the transformation of Neoproterozoic ecosystems affected the way how 2-methylhopanoids were produced in a broader sense.

The dates of the HpnP gene loss event for other marine planktonic cyanobacterial lineages – *i.e.* *Trichodesmium* and *Chrocosphaera/Atelocyanobacterium* clades – were similarly estimated (Extended Data Fig. 2) and none of them was in the Neoproterozoic. Instead, all of the relevant gene loss events seem to have occurred in the Paleo- and Mesoproterozoic, when the

diversification of major cyanobacterial clades proceeded. Hence, the HpnP gene loss in early cyanobacteria may be associated with environmental settings during that time.

Supplementary Note 4: Comparison of the present study with recent previous studies

2-Methylhopanoids have constantly been a hotly debated topic in organic geochemistry due to the importance of their evolutionary implications. Our present study is in part consistent with previous works and also includes novel observations. In this section, three recent studies are individually compared to our present study to illustrate the similarities as well as differences and complement the main text. Our study is built on those previous works, expanding an important essence of individual works – 1) genetics of 2-methylhopanoid biosynthesis, 2) mutualistic relationships of 2-methylhopanoid producers and 3) taxonomic diversity of 2-methylhopanoid producers.

The previous study by Ricci et al. in 2015[1] represents the first attempt to constrain the temporal and taxonomic origin of HpnP and hence is conceptually similar to the genetics part of our present study. The study by Ricci et al. proposed a late origin of HpnP within Alphaproteobacteria and subsequent HGT to Cyanobacteria through phylogenetic analyses. At the time of its publication, the available genetic data were limited, compared to today, and HpnP was nearly confined to Cyanobacteria and Alphaproteobacteria, except only for a single sequence from Acidobacteria. Also, the phylogenetic analyses used proteins that were only distantly related to HpnP as the outgroup. Hence, the resulting tree topology, in particular the rooting of the cyanobacterial clade, was potentially influenced by long-branch attraction. In contrast, our study utilizes a more comprehensive genetic dataset, including close HpnP homologs from more than ten different bacterial phyla (Fig. 1) and also proteins that potentially represent intermediate states between HpnP and HpnP-like proteins (Fig. 2). Hence, the possibility of long-branch attraction is substantially lowered and the observed tree topology is more likely to reflect the actual evolutionary history of HpnP. Additionally, the tree topology was examined using two different methods (maximum likelihood and Bayesian inferences), while the previous study used only the former. The tree topology is broadly consistent between the two inferences and the support for major nodes is generally high. Hence, the HpnP tree topology is more robustly supported in the present study. Our results suggest that the direct HpnP gene transfer between Alphaproteobacteria and Cyanobacteria is unlikely, contrary to the previous inference.

A more recent study by Elling et al. in 2020[9] examined the vitamin B_{12} dependency of 2-methylhopanoid production and introduced the concept of symbiosis between 2-methylhopanoid producers and other microorganisms, particularly focusing on Alphaproteobacteria. This study greatly expanded our understanding of 2-methylhopanoid biosynthesis and proposed a direct link between alphaproteobacterial 2-methylhopanoid production and a particular metabolic network (nitrogen cycle). The study highlighted 2-methylhopanoid production as part of a larger metabolic network in association with geological perturbations. However, the applicability of the proposed specific relationship to the entire Phanerozoic and even the Precambrian was not clear. Our study proposes a more generalized mutualistic relationship between 2-methylhopanoid producers and other organisms, particularly eukaryotic algae. Our mutualistic model has a broader applicability throughout the Phanerozoic and additionally at least the terminal Proterozoic. The model is in fact complementary to the previously proposed relationship of the 2-MHI and nitrogen cycle excursions and also any additional relationships that are not taken into account thus far, reflecting the complex multifaceted nature of ecosystems on Earth.

Another recent study by Naafs et al. in 2021[10] provided great details about potential marine 2-methylhopanoid producers by thorough literature examinations of cultured cyanobacterial and alphaproteobacterial species that were collected from marine environments. This study discussed three lineages of potential 2-methylhopanoid producers in association with their ecological relevance, including their involvement in the nitrogen cycle, as also discussed by Elling et al., 2020[9]. While the study by Naafs et al. deepened our understanding of 2-methylhopanoids in modern organisms, the dataset was restricted to cultured species that were experimentally confirmed to produce hopanoids. It is not realistic to assume only a few specific lineages to explain the entire 2-methylhopanoid production throughout Earth's history. None of the three lineages seems to have a long enough history to elucidate the marine 2-methylhopanoid production for the entire Phanerozoic and older periods.

Our current study includes uncultured species and thus the data size is substantially larger than in the previous study. Several additional lineages of marine 2-methylhopanoid producers were found and some of them likely have a Precambrian origin. In particular, marine (coastal) species were found in many HpnP-containing cyanobacterial lineages and are thus more common than previously thought. The mere presence of HpnP does not necessarily mean the regular production of 2-methylhopanoids since many genes are expressed only under specific conditions[11]. However, Naafs et al., 2021 in fact showed that HpnP-containing species generally produce 2-methylhopanoids under normal lab conditions. Hence, it is reasonable to infer that HpnP-containing marine cyanobacteria contribute to at least the proximal marine 2-methylhopanoid record. In our dataset, marine HpnP-containing alphaproteobacteria are still limited, relative to marine cyanobacteria, but metagenomic data similarly implies the presence of additional marine lineages. Halotolerant species may also have contributed to marine 2-methylhopanoids, as suggested by *Nitrobacter* and *Rhodopseudomonas* (see Supplementary Note 5). In summary, our present study expands the argument about the taxonomic diversity of 2-methylhopanoids and urges further examinations through both computational and experimental approaches.

Supplementary Note 5: Marine 2-methylhopanoid producers in Cyanobacteria and Alphaproteobacteria

In a previous study, only three marine species – one from Cyanobacteria and two from Alphaproteobacteria – were described as marine 2-methylhopanoid producers: *filamentous cyanobacterium ESFC-1*, *Methylobacterium salsuginis* and *Nitrobacter sp. Nb-311A*[10]. Ancestors of *filamentous cyanobacterium ESFC-1* were attributed to the production of Precambrian marine 2-methylhopanoids, while ancestors of *Methylobacterium* were attributed to the production of Phanerozoic marine 2-methylhopanoids. The marine origin of *Nitrobacter sp. Nb-311A* was questioned because its 16S rRNA gene sequence is 100% identical to that of the soil isolate *N. winogradskyi*[12], but *Nitrobacter* is generally halotolerant and thus presumably can live in both terrestrial and marine environments[12]. In our study, HpnP was found to be ubiquitous in the genus and hence *Nitrobacter* has the potential to contribute to the marine 2-methylhopanoid record. In contrast, *M. salsuginis* is not halotolerant and thus cannot grow under saline conditions, despite that the species was isolated from seawater[13]. Hence, it is not clear if *M. salsuginis* is truly ecologically significant in marine environments.

In any case, those two species in *Nitrobacter* and *Methylobacterium* cluster among a large number of non-marine relatives in the same genus and nearby genera. Thus, it is most likely that those marine species evolved from a non-marine ancestor, while retaining HpnP. The transition from non-marine to marine was only after the divergence of individual genera, at the oldest around 300 Ma[14]. Hence, *Nitrobacter* and *Methylobacterium* may explain some recent 2-MHI excursion events, including OAEs, but not the entire Phanerozoic 2-methylhopanoid production. Similarly, it is unlikely that the single lineage of *filamentous cyanobacterium ESFC-1*, which evolved within the family *Oscillatoriaceae*[15], explains all Precambrian 2-methylhopanoids.

In our current study, more than ten additional marine species were found to possess HpnP, by exploring NCBI and JGI databases. These species were not included in the previous study[10] that focused on species that were experimentally confirmed to produce 2-methylhopanoids. For Cyanobacteria, 11 species from 9 different families were retrieved, including species that have only metagenomic information (Supplementary Table 5). All of them are derived from near-shore environments. This is consistent with a previous metagenome study of stromatolites from the hypersaline Shark Bay in western Australia, where predominantly cyanobacterial HpnP were detected[16]. These marine species are taxonomically diverse and distributed in both early- and late-branching clades. Some of the HpnP are possibly the direct descendants of ancestral HpnP in the common ancestor of Cyanobacteria. For instance, HpnP from two unicellular marine species *Aphanocapsa montana BDHKU210001* and *Acaryochloris sp. RCC1774* (*A. thomasi*)[17,18] are closely related to each other and are in fact consistent with the cyanobacterial species relationship (Fig. 3). The lineage that encompasses *Aphanocapsa* and *Acaryochloris* diverged earlier than the lineage that contains the marine planktonic *Prochlorococcus*/*Synechococcus* clade[6,19]. The genera *Acaryochloris* and *Aphanocapsa* include both marine and freshwater species[20,21] and hence it is not clear if these two lineages adapted to marine environments independently or alternatively the common ancestor of *Aphanocapsa* and *Acaryochloris* was already a marine species. In either case, it seems plausible that 2-methylhopanoid production by marine cyanobacteria was present at a relatively early stage of cyanobacterial evolution. In turn, the order Oscillatoriales contains the largest number (six) of marine HpnP-containing species across four families within the order (Supplementary Table 5). Oscillatoria-like filamentous microfossils have been found in ~1.9 Ga Gunflint cherts[22] and thus HpnP in this clade may also have contributed to Precambrian 2-methylhopanoid productions.

It is noted that hopanoid production in general is distributed mostly in terrestrial species (*i.e.* living in freshwater, soil and endolithic environments), as also observed in earlier studies[5]. Among 70 species that possess SC on our complete genome list, only five are derived from marine environments, although they include important nitrogen fixers – *i.e. Atelocyanobacterium thalassa* and *Trichodesmium erythraeum* (Supplementary Table 5). None of the five species contains HpnP. In contrast, marine HpnP that are described above are all derived from draft genome data. Hence, the near absence of marine HpnP in previous studies seems to be due to the limited number of marine cyanobacteria in the datasets, while HpnP is in fact distributed more widely than previously thought within marine cyanobacteria. Further corroborations with environmental DNA studies and also experimental validations of 2-methylhopanoid production for individual marine species would be important to more confidently interpret geological 2-methylhopanes.

For Alphaproteobacteria, *Rhodopseudomonas palustris*, which is usually associated with freshwater environments[23], is distributed also in coastal marine environments[24,25]. *R. palustris* is a halotolerant species, similar to *Nitrobacter* (both in the same family *Nitrobacteraceae*), and thus

can thrive in both freshwater and marine environments. In fact, the transition between marine and non-marine habitats seems to be common and occurred multiple times independently in both Cyanobacteria and Alphaproteobacteria[5,26]. Hence, it is plausible that a small, but certain fraction of Cyanobacteria and (from the late Neoproterozoic onwards) Alphaproteobacteria may have constantly contributed to 2-methylhopanoid production in marine environments, regardless of their normal habitats. Their taxonomic origin was not necessarily identical at individual geological times. Similar to Cyanobacteria, metagenomic data suggest potentially a wider distribution of marine HpnP in Alphaproteobacteria (Supplementary Table 3), as was also suggested in a previous study[5]. In the previous study, 93% (26 out of 28) of the detected marine HpnP sequences were attributed to Alphaproteobacteria[5], demonstrating that HpnP-containing alphaproteobacteria are the dominant 2-methylhopanoid producers in many modern marine environments and may have similarly been important across the Phanerozoic and the late Neoproterozoic after the emergence of this metabolic capacity within the phylum (*i.e.* after 750 Ma according to our analyses). Overall, our results imply that the source of marine 2-methylhopanoids is not necessarily confined only to a few specific lineages that have a cultured representative for both Cyanobacteria and Alphaproteobacteria.

<u>Supplementary Note 6</u>: Syngeneity assessment of Paleoproterozoic 2-methylhopanes from the McArthur Basin, Northern Australia (GR7 and LV09001 cores)

In our current study, the detailed spatial distribution patterns within rock specimens, and hence the syngeneity of 2-methylhopanes, was examined for two Paleoproterozoic drill cores; GR7 and LV09001. Drill core GR7 has long been utilized for paleoecological reconstructions of the Barney Creek Formation (BCF)[27]. A more recently drilled core LV09001 was obtained from the southern McArthur Basin (east of the Emu Fault). In contrast to GR7, where only the upper section of the BCF is thermally well-preserved, LV09001 is thermally well-preserved throughout the entire section of the BCF as well as underlying Teena Dolostone and overlying Reward Dolostone & Lynott Formation. Therefore, LV09001 allows us to extend 2-MHI analysis to a wider stratigraphy and a longer geological time scale. The upper section of the BCF from the GR7 sample exhibits the highest TOC (up to 8%) and the lowest thermal maturity (T_{max} ca. 435-445 °C, HI ca. 500-800 mg hydrocarbons/g organic carbon)[28]. Thermal maturity of the LV09001 samples ranges from immature to early mature (T_{max} ca. 424–438 °C)[29].

Drill core GR7

Drill core GR7 has different thermal maturities between the upper and the lower sections. Only the upper ~200 m section of the core appears thermally suitable for trace biomarker analyses[30], but samples from both sections were analyzed for comparison. Most samples were analyzed by the bulk analysis, while the 45.4 m sample was analyzed by the slice-extraction experiment and the 685.8 m & 869.6 m samples were analyzed by interior-exterior experiments (Supplementary Table 6). The total hopane concentration substantially varies with depth and broadly displays an anticorrelation to the 2-MHI. Hopane-rich samples generally have low 2-MHI values (mostly <3%), except for the 218.1 m sample, while hopane-poor samples generally have higher 2-MHI values (up to 16.7%). The spatial distribution of biomarkers within rock specimens (*i.e.* hopanes and steranes) for the 683.5 m and 869.9 m samples that yield the highest 2-MHI values of 16.7 and 13.2%, respectively, suggest that biomarkers are largely restricted to the sample exterior, while the sample interior is devoid of detectable biomarkers. These observations suggest that the elevated 2-MHI values for hopane-poor bulk samples are likely to reflect contamination overprint that was

probably introduced during drilling, cutting and storage. 2-Methylhopanes in the contaminants generally exhibit high 2-MHI values. In contrast, hopane-rich samples, which are less susceptible to surficial contamination and thus may retain largely syngenetic (methyl)hopane signatures, exhibit low 2-MHI values. In the present study, GR7 samples that were only bulk analyzed were not included in our dataset to minimize uncertainty considering the trace abundances of 2-methylhopanes.

Drill core LV09001
The molecular inventory of core samples from hole LV09001 allows for the first time to investigate the methylhopane distribution in the lower (deeper) section of the BCF as well as underlying Teena Dolostone and overlying Reward Dolostone and Lynott Formation. All samples were separated into interior and exterior portions. The level of surficial contamination in the core was assessed by interior/exterior comparisons on seven samples. Steranes are below the detection limit in nearly all interior and exterior samples, with an exception of trace amounts in a few exteriors. Hopanes are abundant in both the exterior and the interior of samples for all formations. The abundance of hopanes is nearly identical between the exterior and the interior of all analyzed samples. These observations indicate that the level of surficial contamination for LV09001 drill core is very low, unlike (parts of) the GR7 core, and its impact on our biomarker analyses is negligible with nearly all of the detected hopanes being inferred to be syngenetic. 2-MHI values are generally low throughout the core (Supplementary Table 7), regardless of total hopane abundance. The low 2-MHI values for non-contaminated LV09001 samples are consistent with those for hopane-rich GR7 samples that are geographically separated from the LV09001 samples within the McArthur Basin. In the present study, all of the LV09001 samples are included in our dataset.

Supplementary Note 7: Possibility of diagenetic 2-methylhopane production

Apart from biological 2-methylhopanoid production, we addressed the possibility that diagenetically-mediated reactions may lead to the methylation of hopanoids, thereby mimicking biological signatures. For example, 2- and 3-methylsteroids that have been observed in geological records for decades[31] were recently shown to readily form through an abiogenic alkylation process affecting double bonds and/or functional groups in non-alkylated steroid precursors[32]. In the present study, pyrolysis experiments using diplopterol (a major bacterial hopanoid) as well as cholesterol and its saturated equivalent cholestanol (control compounds) were conducted to test the possibility of an abiogenic origin for 2- and 3-methylhopanoids. Whereas cholesterol and cholestanol, which possess a functionalized A-ring, underwent the expected C-2 and C-3 methylations, diplopterol did not generate 2- or 3-methylhopanes (Supplementary Figs. 11−12). Our results suggest that typical bacterial hopanoids, which do not possess a functional group in the A-ring, are unlikely to be subjected to abiogenic C-2 and C-3 methylations and 2-methylhopanes therefore retain their potential to act as ecological markers.

Supplementary Note 8: Depositional setting for marine 2-methylhopanoids

There is a possibility that the apparent low 2-MHI values in the mid-Proterozoic may be due to a mismatch between 2-methylhopanoid production and depositional sites. The distribution of HpnP in marine cyanobacteria is confined to benthic species (Supplementary Table 5 and Supplementary Note 5), and 2-methylhopanoid production by modern ecosystems is in fact observed only in near-shore environments[16,33]. It has similarly been inferred that marine cyanobacteria before the rise of modern planktonic species in the Neoproterozoic, regardless of having HpnP or not, was mostly

benthic[6]. This inference is based on the observation that modern planktonic species are taxonomically limited and the evolution of those species seems not to be older than the Neoproterozoic, although the possibility of the presence of extinct planktonic lineages is not excluded[6]. Considering the likely broader distribution of microbial mats in the Precambrian than in the Phanerozoic[34,35], a substantial proportion of pre-Ediacaran marine 2-methylhopanoids was likely derived from benthic cyanobacterial biomass. Any terrigenous inputs would enhance the abundance of 2-methylhopanoids because HpnP is more abundant in terrestrial species, although there is currently no strong evidence for substantial terrigenous inputs before 850 Ma[36].

Our mid-Proterozoic samples include both shallow and deeper water samples and yet the 2-MHI remains low, regardless of the depositional setting. This suggests no appreciable depositional bias for the mid-Proterozoic 2-MHI record. 2-Methylhopanoids may have been laterally transported from coastal regions to deeper waters *via* the littoral shuttle, as initially proposed for meromictic Mahoney Lake in Canada and hypothesized to have been potentially important early in Earth´s history[37], although the importance of the littoral shuttle throughout Earth history remains largely unconstrained. The emergence of modern pelagic planktonic cyanobacteria (HpnP absent) and the decline of benthic cyanobacteria in post-Cryogenian oceans would reduce the relative abundance of 2-methylhopanoids and thus the 2-MHI. Additionally, aerobic degradation of cyanobacterial biomass by heterotrophic bacteria, which produce mostly only non-methylated hopanoids, would further reduce the 2-MHI. Such a process was likely intensified in more oxic environments and thus in the Phanerozoic. Hence, if the 2-MHI trend were to reflect only cyanobacterial inputs, we would expect higher 2-MHI values in pre-Ediacaran periods, but a decrease towards the Phanerozoic. However, the observed trend shows the opposite. Therefore, the dramatic increase in the 2-MHI more likely reflects the emergence of novel non-cyanobacterial sources (*i.e.* Alphaproteobacteria), although this does not necessarily preclude the involvement of (particularly benthic) cyanobacteria in certain 2-MHI excursion events in the Phanerozoic under specific conditions. The dominance of alphaproteobacterial hpnP in modern marine metagenomes[5] support the inference that Alphaproteobacteria was among the important 2-methylhopanoid producers in many marine environments for much of the Phanerozoic.

<u>Supplementary Note 9</u>: Vitamin B_{12} (cobalamin) dependency of algae

The vitamin B_{12} dependency of plants and algae is complex. For instance, land plants do not require vitamin B_{12} because they can produce methionine using the vitamin B_{12}-independent form of methionine synthase (MetE), while algae have MetE and/or the vitamin B_{12}-dependent form (MetH), depending on species[38]. Red algae retain MetE and additionally MetH in many species, but the majority of green algae have only MetH and thus are vitamin B_{12}-dependent. A selective advantage of having MetH instead of MetE is a substantially higher efficiency of methionine biosynthesis by MetH (x50 times)[39,40]. MetE and MetH do not share a sequence similarity and are inferred to have evolved independently[41]. However, both enzymes are taxonomically widespread in Archaeplastida (land plants + algae) and phylogenetic analyses suggest that the individual common ancestors of red algae, green algae and the entire Archaeplastida clade had both forms of enzymes, except for the common ancestor of land plants that likely had only MetE[38]. Therefore, the MetE or MetH gene was selectively lost in individual algal lineages during algal evolution.

Vitamin B_{12}-dependent green algae rely on the uptake of this nutrient produced by symbiotic bacteria or archaea since no eukaryotes can biosynthesize vitamin B_{12} *de novo*[42]. There are two

known pathways to biosynthesize the corrin ring of vitamin B_{12} – oxygen-independent and oxygen-dependent pathways[43]. The oxygen-independent pathway is mainly present in anaerobic lineages such as Clostridia (Firmicutes) and Desulfobacteria (Deltaproteobacteria), but is also found in some aerobic lineages such as Cyanobacteria and Thaumarchaeota[43]. The oxygen-independent pathway may have been important for Cyanobacteria to endure oxygen stress at the onset of oxygenic photosynthesis[44]. In contrast, the oxygen-dependent pathway is mostly confined to some late-branching taxa of Alphaproteobacteria (*e.g.* Hypomicrobiales, Rhodobacterales) and Gammaproteobacteria (*e.g.* Methylococcales, Pseudomonadales)[43]. However, the major contributors of vitamin B_{12} to algae in modern environments are those bacteria that utilize the oxygen-dependent pathway, while vitamin B_{12} production in deeper waters is largely contributed by Thaumarchaeota[44]. Cyanobacteria produces pseudocobalamin, instead of vitamin B_{12}, because Cyanobacteria utilizes a different ligand that attaches to the corrin ring. Thus, Cyanobacteria cannot directly contribute to the vitamin B_{12} pool, although a small fraction of algae are known to possess enzymes to convert pseudocobalamin to vitamin B_{12}[39].

The wide occurrence of vitamin B_{12} dependency in green algae reflects an ancient origin of the algal symbiosis with vitamin B_{12}-producing microbes and the accompanied MetE gene loss[42]. However, the distribution of vitamin B_{12} dependency is not reflected in the phylogeny of green algae. Similarly, microbial taxa that are known to have a symbiotic relationship with algae are not particularly close to each other. Thus, the dependency between green algae and bacteria evolved many times independently in different algal and microbial lineages, including both freshwater and marine species[45]. The symbiosis between green algae and bacteria is not strictly a co-evolutionary relationship, but is characterized by more loose and broad interactions on occasion. For instance, the green alga *Chlamydomonas nivalis* and the alphaproteobacterium *Mesorhizobium loti* (Hypomicrobiales) establish a mutualistic relationship, in exchange of carbon source and vitamin B_{12}, under laboratory culture conditions[46]. *M. loti* is a rhizobacterium that is found in root nodules in native environments[47] and thus the symbiosis with *C. nivalis* is a non-natural relationship induced by the specific lab condition. Also, *Chlamydomonas reinhardtii* has a mutualistic relationship with multiple bacterial taxa, such as *Rhizobium*, *Shinella*, *Pseudomonas* and *Flavobacterium* (Hypomicrobiales is the most abundant)[48]. Hence, green algae obtain vitamin B_{12} from a variety of microbial sources that are available at different times and locations and do not require a fixed relationship with specific microbial partners. In turn, vitamin B_{12}-producing bacteria likely obtain carbon sources from various green algae among others.

The expansion of 2-methylhopanoid-producing alphaproteobacteria in association with vitamin B_{12} production currently remains speculative because a symbiotic relationship between marine algae and HpnP-containing species has not been directly observed in modern environments thus far. For instance, Hyphomicrobiales is more frequently associated with terrestrial or freshwater environments (*e.g.* lichens) than with oceans[49]. However, metagenomic surveys predict the presence of both Hyphomicrobiales and alphaproteobacterial HpnP in marine settings[5,50]. Also, some Hyphomicrobiales species are in fact associated with marine algae[51,52]. Studies that focus on the taxonomic diversity of the modern marine algal-bacterial relationship would be beneficial to shed more light on the co-evolution of vitamin B_{12}-auxotrophic algae and bacterial donors[53]. However, it is noted that the taxonomic affiliation of dominant vitamin B_{12} producers in marine environments is not a critical factor to associate HpnP with vitamin B_{12}. As described above, the symbiosis between eukaryotic algae and bacteria seems to be not taxon-specific, but the sum of direct and indirect interactions between a group of vitamin B_{12}-auxotrophs and producers. A large

community of vitamin B_{12}-producing bacteria (in particular, Alpha- and Gammaproteobacteria) collectively support the growth of vitamin B_{12}-auxotrophic eukaryotic algae. Within the community, a fraction would represent 2-methylhopanoid producers. Yet, as shown by the abundance of *Rhizobium* sp. in *C. reinhardtii*[48], Hyphomicrobiales may be a major vitamin B_{12} source for at least some green algae.

Supplementary Information references

1 Ricci, J. N., Michel, A. J. & Newman, D. K. Phylogenetic analysis of HpnP reveals the origin of 2-methylhopanoid production in Alphaproteobacteria. *Geobiology* **13**, 267-277 (2015).

2 Muñoz-Gómez, S. A. et al. An updated phylogeny of the Alphaproteobacteria reveals that the parasitic Rickettsiales and Holosporales have independent origins. *eLife* **8**, e42535 (2019).

3 Hoshino, Y. & Gaucher, E. A. Evolution of bacterial steroid biosynthesis and its impact on eukaryogenesis. *Proc. Natl. Acad. Sci. USA* **118**, e2101276118 (2021).

4 Kulkarni, G. et al. Specific Hopanoid Classes Differentially Affect Free-Living and Symbiotic States of *Bradyrhizobium diazoefficiens*. *mBio* **6**:e01251-15 (2015).

5 Ricci, J. N. et al. Diverse capacity for 2-methylhopanoid production correlates with a specific ecological niche. *ISME J.* **8**, 675-684 (2014).

6 Sánchez-Baracaldo, P. Origin of marine planktonic cyanobacteria. *Sci. Rep*. **5**, 17418 (2015).

7 Sánchez-Baracaldo, P., Bianchini, G., Di Cesare, A., Callieri, C. & Chrismas, N. A. M. Insights Into the Evolution of Picocyanobacteria and Phycoerythrin Genes (mpeBA and cpeBA). *Front. Microbiol*. **10**:45 (2019).

8 Hirose, Y. et al. Diverse Chromatic Acclimation Processes Regulating Phycoerythrocyanin and Rod-Shaped Phycobilisome in Cyanobacteria. *Mol. Plant* **12**, 715-725 (2019).

9 Elling, F. J. et al. Vitamin B_{12}-dependent biosynthesis ties amplified 2-methylhopanoid production during oceanic anoxic events to nitrification. *Proc. Natl. Acad. Sci. USA* **117**, 32996 (2020).

10 Naafs, B. D. A., Bianchini, G., Monteiro, F. M. & Sánchez-Baracaldo, P. The occurrence of 2-methylhopanoids in modern bacteria and the geological record. *Geobiology* **20**, 41-59 (2022).

11 Yuan, Y. et al. Efficient exploration of terpenoid biosynthetic gene clusters in filamentous fungi. *Nat. Catal.* **5**, 277-287 (2022).

12 Starkenburg Shawn, R. et al. Complete Genome Sequence of *Nitrobacter hamburgensis* X14 and Comparative Genomic Analysis of Species within the Genus *Nitrobacter*. *Appl. Environ. Microbiol.* **74**, 2852-2863 (2008).

13 Green, P. N. & Ardley, J. K. Review of the genus *Methylobacterium* and closely related organisms: a proposal that some *Methylobacterium* species be reclassified into a new genus, *Methylorubrum* gen. nov. *Int. J. Syst. Evol. Microbiol*. **68**, 2727-2748 (2018).

14 Wang, S., Meade, A., Lam, H.-M. & Luo, H. Evolutionary Timeline and Genomic Plasticity Underlying the Lifestyle Diversity in *Rhizobiales*. *mSystems* **5**, e00438-00420 (2020).

15 Everroad, R. C. et al. Permanent draft genome of strain ESFC-1: ecological genomics of a newly discovered lineage of filamentous diazotrophic cyanobacteria. *Stand. Genom. Sci.* **11**, 53 (2016).
16 Garby, T. J., Walter, M. R., Larkum, A. W. D. & Neilan, B. A. Diversity of cyanobacterial biomarker genes from the stromatolites of Shark Bay, Western Australia. *Environ. Microbiol.* **15**, 1464-1475 (2013).
17 Bhattacharyya, S. et al. Draft Genome Sequence of Exopolysaccharide-Producing Cyanobacterium *Aphanocapsa montana* BDHKU 210001. *Genome Announc*. **3**, e00057-00015 (2015).
18 Partensky, F. et al. A novel species of the marine cyanobacterium *Acaryochloris* with a unique pigment content and lifestyle. *Sci. Rep.* **8**, 9142 (2018).
19 Moore, K. R. et al. An Expanded Ribosomal Phylogeny of Cyanobacteria Supports a Deep Placement of Plastids. *Front. Microbiol*. **10**:1612 (2019).
20 Komárek, J. in Freshwater Algae of North America (eds John D. Wehr & Robert G. Sheath) 59-116 (Academic Press, 2003).
21 Zhang, Z.-C. et al. Widespread occurrence and unexpected diversity of red-shifted chlorophyll producing cyanobacteria in humid subtropical forest ecosystems. *Environ. Microbiol.* **21**, 1497-1510 (2019).
22 Wacey, D. et al. Nanoscale analysis of pyritized microfossils reveals differential heterotrophic consumption in the ~1.9-Ga Gunflint chert. *Proc. Natl. Acad. Sci. USA* **110**, 8020-8024 (2013).
23 Jiao, Y., Kappler, A., Croal Laura, R. & Newman Dianne, K. Isolation and Characterization of a Genetically Tractable Photoautotrophic Fe(II)-Oxidizing Bacterium, *Rhodopseudomonas palustris* Strain TIE-1. *Appl. Environ. Microbiol.* **71**, 4487-4496 (2005).
24 Herbert, R. A. Isolation and Identification of Photosynthetic Bacteria (Rhodospirillaceae) from Antarctic Marine and Freshwater Sediments. *J. Appl. Bacteriol*. **41**, 75-80 (1976).
25 Harwood, C. S. *Rhodopseudomonas palustris*. *Trends Microbiol*. **30**, 307-308 (2022).
26 Sánchez-Baracaldo, P., Raven, J. A., Pisani, D. & Knoll, A. H. Early photosynthetic eukaryotes inhabited low-salinity habitats. *Proc. Natl. Acad. Sci. USA* **114**, E7737-E7745 (2017).
27 Brocks, J. J. et al. Biomarker evidence for green and purple sulphur bacteria in a stratified Palaeoproterozoic sea. *Nature* **437**, 866-870 (2005).
28 Summons, R. E., Powell, T. G. & Boreham, C. J. Petroleum geology and geochemistry of the Middle Proterozoic McArthur Basin, Northern Australia: III. Composition of extractable hydrocarbons. *Geochim. Cosmochim. Acta* **52**, 1747-1763 (1988).
29 Vinnichenko, G., Jarrett, A. J. M., van Maldegem, L. M. & Brocks, J. J. Substantial maturity influence on carbon and hydrogen isotopic composition of n-alkanes in sedimentary rocks. *Org. Geochem*. **152**, 104171 (2021).
30 Lee, C. & Brocks, J. J. Identification of carotane breakdown products in the 1.64billion year old Barney Creek Formation, McArthur Basin, northern Australia. *Org. Geochem*. **42**, 425-430 (2011).
31 Summons, R. E. & Capon, R. J. Fossil steranes with unprecedented methylation in ring-A. *Geochim. Cosmochim. Acta* **52**, 2733-2736 (1988).
32 van Maldegem, L. M. et al. Geological alteration of Precambrian steroids mimics early animal signatures. *Nat. Ecol. Evol.* **5**, 169–173 (2021).

33 Allen, M. A., Neilan, B. A., Burns, B. P., Jahnke, L. L. & Summons, R. E. Lipid biomarkers in Hamelin Pool microbial mats and stromatolites. *Org. Geochem*. **41**, 1207-1218 (2010).
34 Gehling, J. G. Microbial mats in terminal Proterozoic siliciclastics; Ediacaran death masks. *PALAIOS* **14**, 40-57 (1999).
35 Pawlowska, M. M., Butterfield, N. J. & Brocks, J. J. Lipid taphonomy in the Proterozoic and the effect of microbial mats on biomarker preservation. *Geology* **41**, 103-106 (2013).
36 Knauth, L. & Kennedy, M. The late Precambrian greening of the Earth. *Nature* **460**, 728-732 (2009).
37 Bovee, R. J. & Pearson, A. Strong influence of the littoral zone on sedimentary lipid biomarkers in a meromictic lake. *Geobiology* **12**, 529-541 (2014).
38 Helliwell, K. E., Wheeler, G. L., Leptos, K. C., Goldstein, R. E. & Smith, A. G. Insights into the Evolution of Vitamin B_{12} Auxotrophy from Sequenced Algal Genomes. *Mol. Biol. Evol.* **28**, 2921-2933 (2011).
39 Helliwell, Katherine E. et al. Cyanobacteria and Eukaryotic Algae Use Different Chemical Variants of Vitamin B_{12}. *Curr. Biol*. **26**, 999-1008 (2016).
40 Mordukhova Elena, A. & Pan, J.-G. Evolved Cobalamin-Independent Methionine Synthase (MetE) Improves the Acetate and Thermal Tolerance of Escherichia coli. *Appl. Environ. Microbiol.* **79**, 7905-7915 (2013).
41 Deobald, D., Hanna, R., Shahryari, S., Layer, G. & Adrian, L. Identification and characterization of a bacterial core methionine synthase. *Sci. Rep*. **10**, 2100 (2020).
42 Croft, M. T., Lawrence, A. D., Raux-Deery, E., Warren, M. J. & Smith, A. G. Algae acquire vitamin B12 through a symbiotic relationship with bacteria. *Nature* **438**, 90-93 (2005).
43 Shelton, A. N. et al. Uneven distribution of cobamide biosynthesis and dependence in bacteria predicted by comparative genomics. *ISME J.* **13**, 789-804 (2018).
44 Heal, K. R. et al. Two distinct pools of B_{12} analogs reveal community interdependencies in the ocean. *Proc. Natl. Acad. Sci. USA* **114**, 364-369 (2017).
45 Grant, M. A. A., Kazamia, E., Cicuta, P. & Smith, A. G. Direct exchange of vitamin B_{12} is demonstrated by modelling the growth dynamics of algal–bacterial cocultures. *ISME J.* **8**, 1418-1427 (2014).
46 Kazamia, E. et al. Mutualistic interactions between vitamin B_{12}-dependent algae and heterotrophic bacteria exhibit regulation. *Environ. Microbiol*. **14**, 1466-1476 (2012).
47 Jarvis, B. D. W., Pankhurst, C. E. & Patel, J. J. *Rhizobium loti*, a New Species of Legume Root Nodule Bacteria. *Int. J. Syst. Evol. Microbiol*. **32**, 378-380 (1982).
48 Kim, B.-H., Ramanan, R., Cho, D.-H., Oh, H.-M. & Kim, H.-S. Role of *Rhizobium*, a plant growth promoting bacterium, in enhancing algal biomass through mutualistic interaction. *Biomass Bioenergy* **69**, 95-105 (2014).
49 Erlacher, A. et al. Rhizobiales as functional and endosymbiontic members in the lichen symbiosis of *Lobaria pulmonaria* L. *Front. Microbiol*. **6**:53 (2015).
50 Yilmaz, P., Yarza, P., Rapp, J. Z. & Glöckner, F. O. Expanding the World of Marine Bacterial and Archaeal Clades. *Front. Microbiol*. **6**:1524 (2016).
51 Barott, K. L. et al. Microbial diversity associated with four functional groups of benthic reef algae and the reef-building coral *Montastraea annularis*. *Environ. Microbiol*. **13**, 1192-1204 (2011).
52 Wu, Z., Yang, X., Lin, S., Lee, W. H. & Lam, P. K. S. A *Rhizobium* bacterium and its population dynamics under different culture conditions of its associated toxic dinoflagellate *Gambierdiscus balechii*. *Mar. Life Sci. Technol*. **3**, 542-551 (2021).

53 Ramanan, R., Kim, B.-H., Cho, D.-H., Oh, H.-M. & Kim, H.-S. Algae–bacteria interactions: Evolution, ecology and emerging applications. *Biotechnol. Adv*. **34**, 14-29 (2016).
54 Williams, M. A Lipid Biomarker Investigation of Organic Matter Sources and Methane Cycling in Alaskan Thaw Lake Sediments, *Doctoral dissertation*, University of California Riverside, (2012).
55 Kuypers, M. M. M., van Breugel, Y., Schouten, S., Erba, E. & Damsté, J. S. S. N_2-fixing Cyanobacteria supplied nutrient N for Cretaceous oceanic anoxic events. *Geology* **32**, 853-856 (2004).
56 Wang, G., Wang, T. G., Simoneit, B. R. T., Zhang, L. & Zhang, X. Sulfur rich petroleum derived from lacustrine carbonate source rocks in Bohai Bay Basin, East China. *Org. Geochem.* **41**, 340-354 (2010).
57 Schaefer, B. et al. Microbial life in the nascent Chicxulub crater. *Geology* **48**, 328-332 (2020).
58 Castro, J. M. et al. Complex and protracted environmental and ecological perturbations during OAE 1a - Evidence from an expanded pelagic section from south Spain (Western Tethys). *Glob. Planet. Change* **183**, 103030 (2019).
59 Stüeken, E. E. et al. Effects of pH on redox proxies in a Jurassic rift lake: Implications for interpreting environmental records in deep time. *Geochim. Cosmochim. Acta* **252**, 240-267 (2019).
60 Saito, R. et al. Secular changes in environmental stresses and eukaryotes during the Early Triassic to the early Middle Triassic. *Palaeogeogr. Palaeocl*. **451**, 35-45 (2016).
61 Xia, L. et al. Coupling of paleoenvironment and biogeochemistry of deep-time alkaline lakes: A lipid biomarker perspective. *Earth-Sci. Rev*. **213**, 103499 (2021).
62 Słowakiewicz, M. et al. Shale-gas potential of the Mid-Carboniferous Bowland-Hodder Unit in the Cleveland Basin (Yorkshire), Central Britain. *J. Pet. Geol.* **38**, 59-75 (2015).
63 Lengger, S. K., Melendez, I. M., Summons, R. E. & Grice, K. Mudstones and embedded concretions show differences in lithology-related, but not source-related biomarker distributions. *Org. Geochem*. **113**, 67-74 (2017).
64 Spaak, G. et al. Environmental conditions and microbial community structure during the Great Ordovician Biodiversification Event; a multi-disciplinary study from the Canning Basin, Western Australia. *Glob. Planet. Change* **159**, 93-112 (2017).
65 Lee, C. et al. Carbon isotopes and lipid biomarkers from organic-rich facies of the Shuram Formation, Sultanate of Oman. *Geobiology* **11**, 406-419 (2013).
66 Parfenova, T. M. First data on methylhopanes in Lower Cambrian organic matter of the Siberian platform. *Dokl. Earth Sci.* **475**, 775-779 (2017).
67 Grosjean, E., Love, G. D., Kelly, A. E., Taylor, P. N. & Summons, R. E. Geochemical evidence for an Early Cambrian origin of the 'Q' oils and some condensates from north Oman. *Org. Geochem*. **45**, 77-90 (2012).
68 Stolper, D. A. et al. Paleoecology and paleoceanography of the Athel silicilyte, Ediacaran–Cambrian boundary, Sultanate of Oman. *Geobiology* **15**, 401-426 (2017).

69 Grosjean, E., Love, G. D., Stalvies, C., Fike, D. A. & Summons, R. E. Origin of petroleum in the Neoproterozoic–Cambrian South Oman Salt Basin. *Org. Geochem.* **40**, 87-110 (2009).

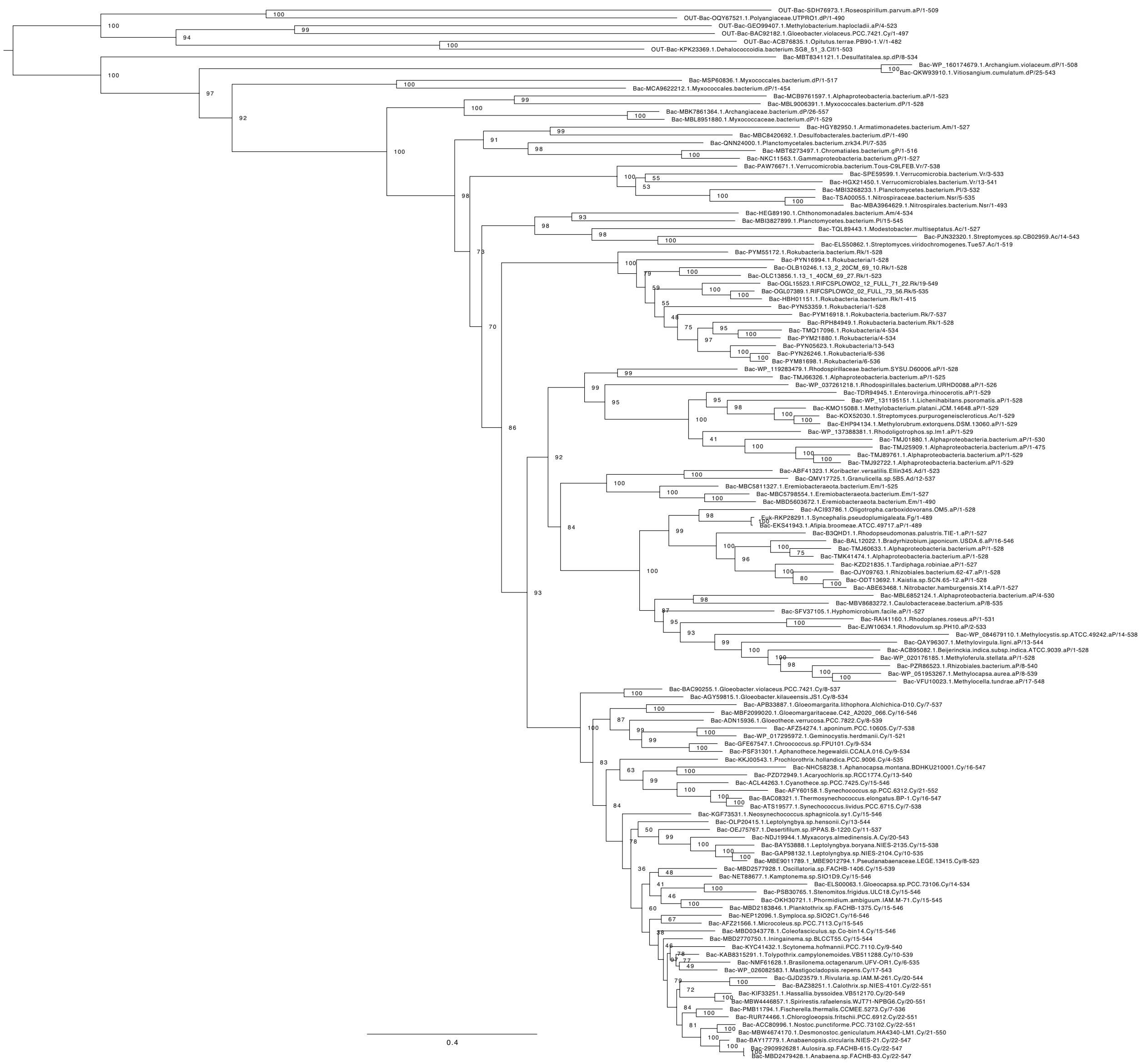

Supplementary Fig. 1. Maximum likelihood phylogenetic tree of HpnP. The figure is identical to Fig. 2 in the main text. The scale bar represents 0.4 amino acid replacements per site per unit evolutionary time. See Supplementary Data 1 for the tree data and Supplementary Data 4 for the sequence alignment.

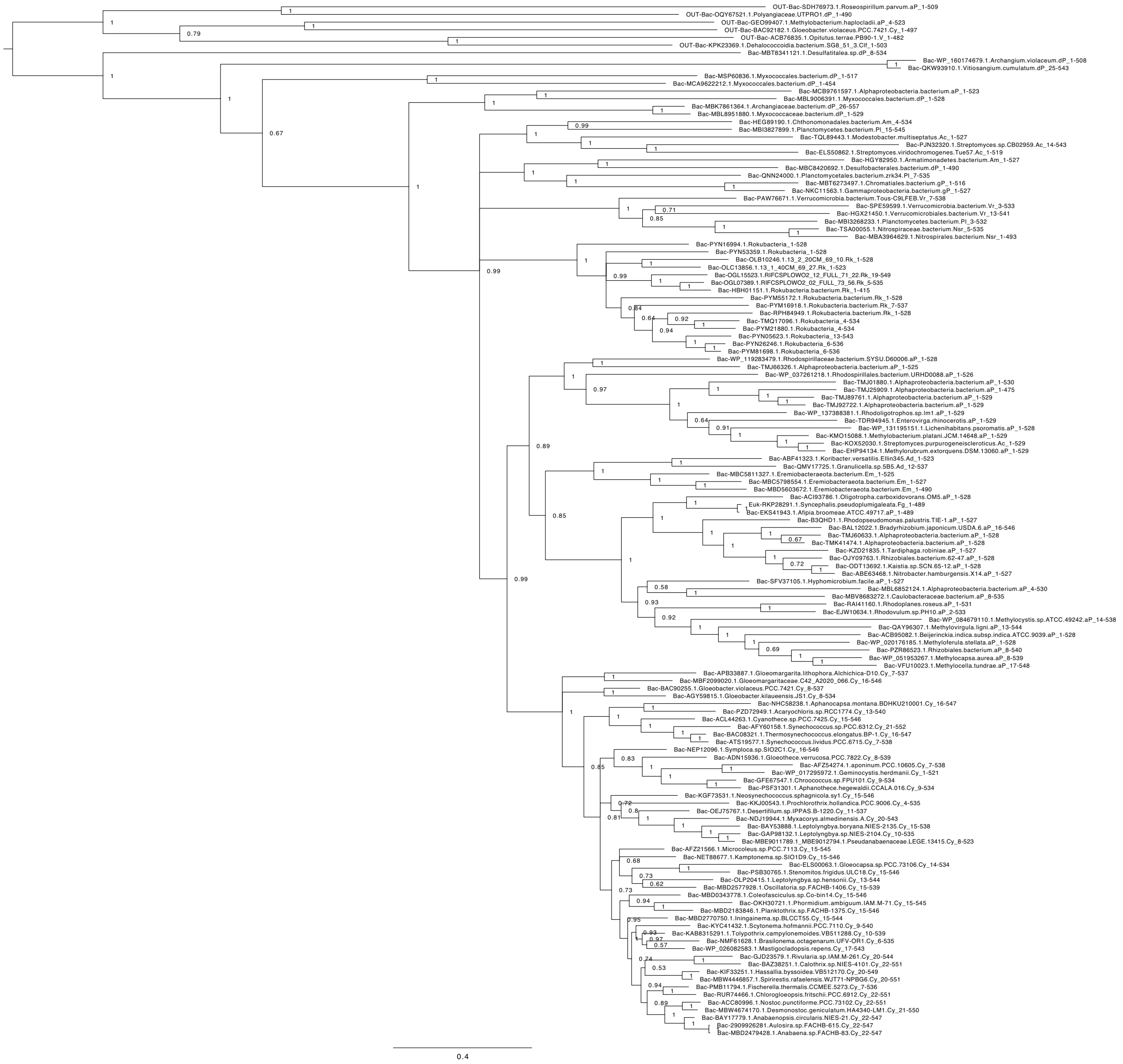

Supplementary Fig. 2. Bayesian phylogenetic tree of HpnP. The scale bar represents 0.4 amino acid replacements per site per unit evolutionary time. See Supplementary Data 2 for the tree data.

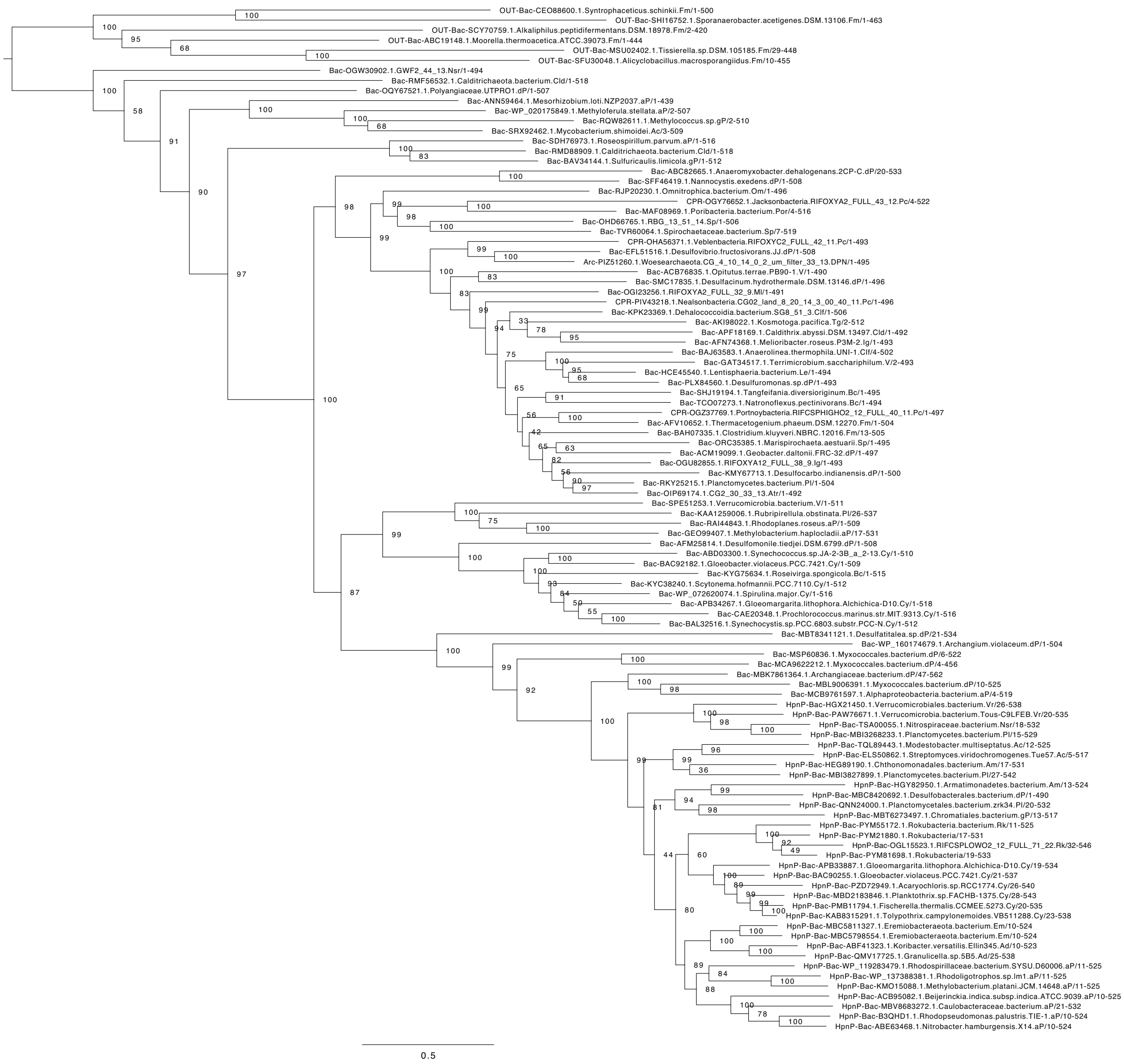

Supplementary Fig. 3. Maximum likelihood tree of class B radical SAM methyltransferases (HpnP homologs). The figure is identical to Fig. 2 insert. The scale bar represents 0.5 amino acid replacements per site per unit evolutionary time. See Supplementary Data 3 for the tree data and Supplementary Data 5 for the sequence alignment.

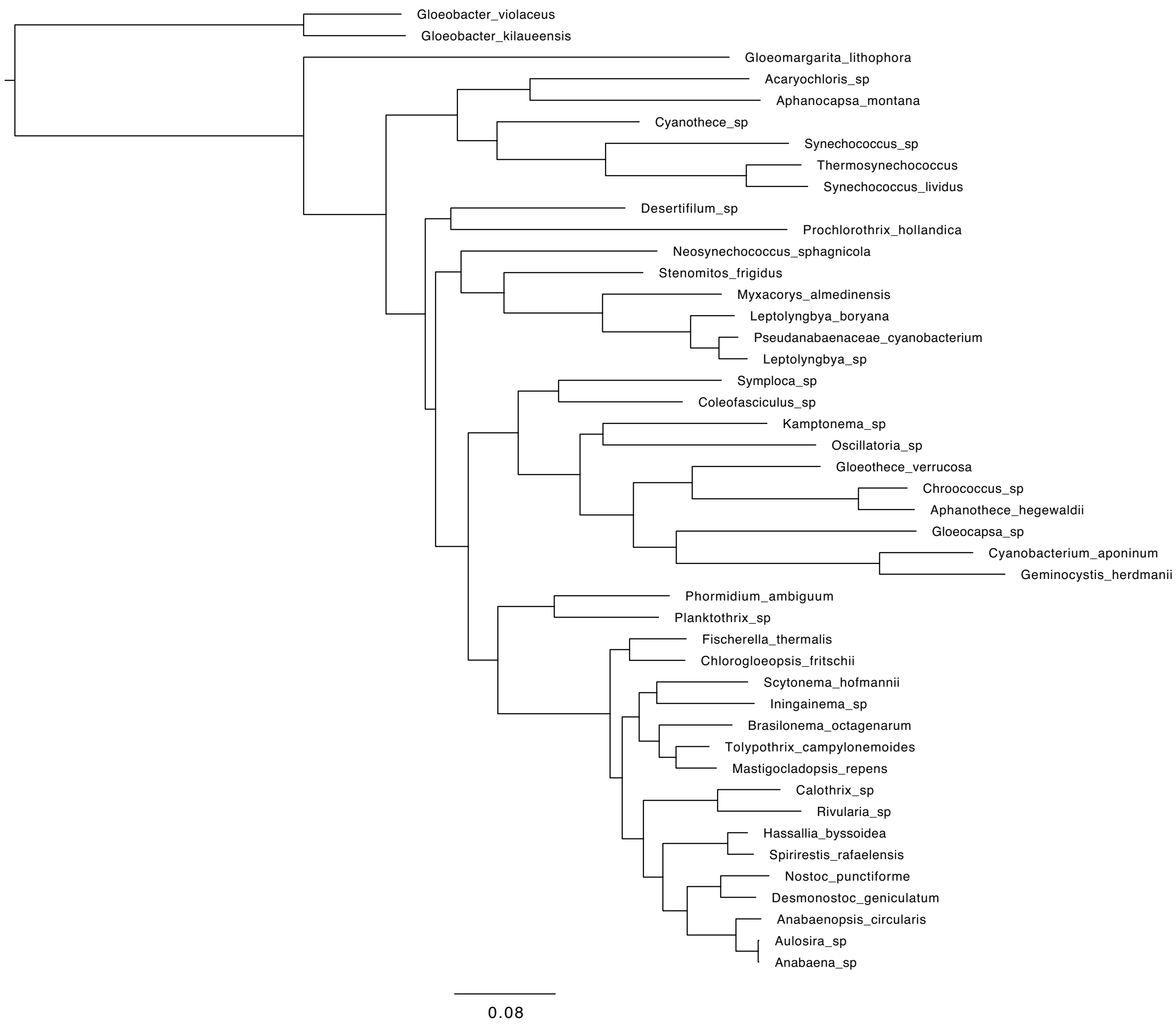

Supplementary Fig. 4. Species tree of 44 HpnP-containing cyanobacteria. The scale bar represents 0.08 amino acid replacements per site per unit evolutionary time.

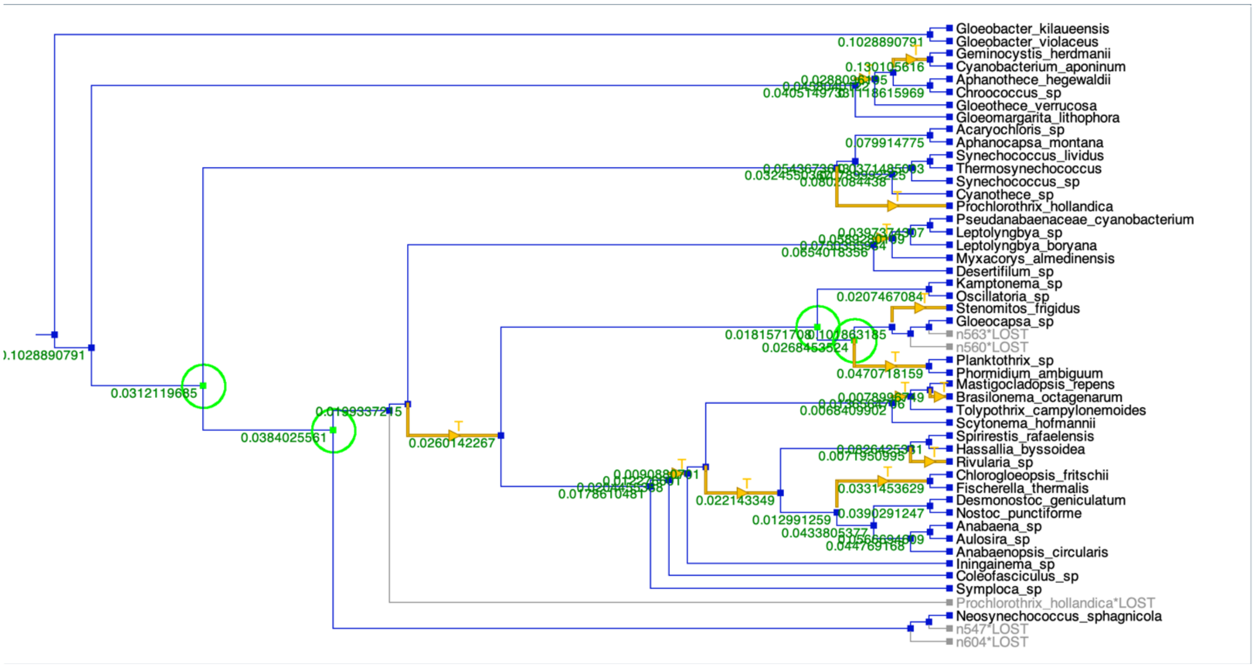

Supplementary Fig. 5. The comparison of cyanobacterial HpnP tree (Fig. 3) and the species tree (Supplementary Fig. 4) by Notung. The label T indicates horizontal gene transfer. The values at individual nodes indicate branch lengths.

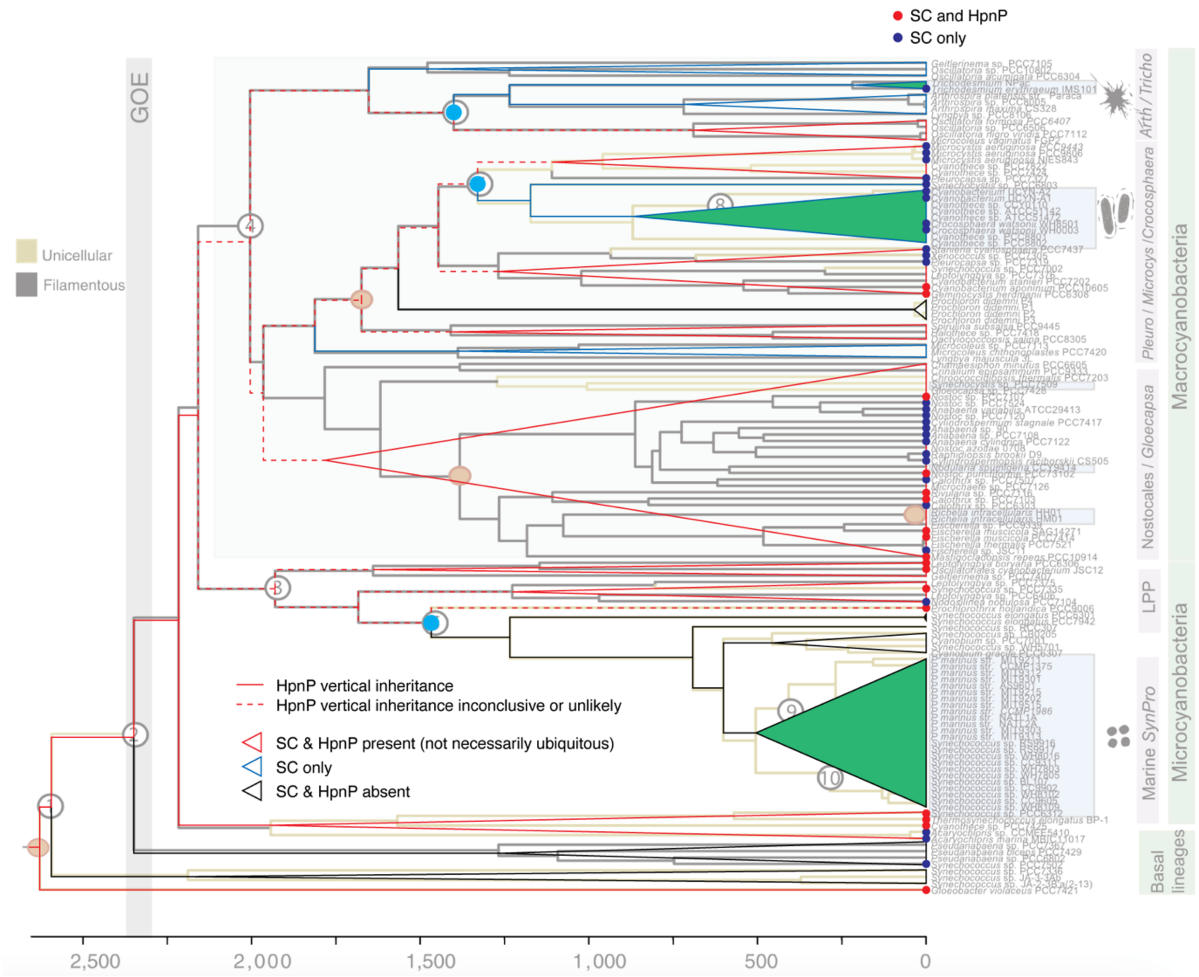

Supplementary Fig. 6. Published dated phylogeny of Cyanobacteria[6] and the superimposition of the simplified tree that is used in Extended Data Fig. 2. In Sánchez-Baracaldo, 2015, relaxed molecular clock analyses were performed utilizing this concatenated tree based on the nucleotide sequences of 135 universally conserved single-copy genes and two ribosomal RNAs. Further details are referred to the original study.

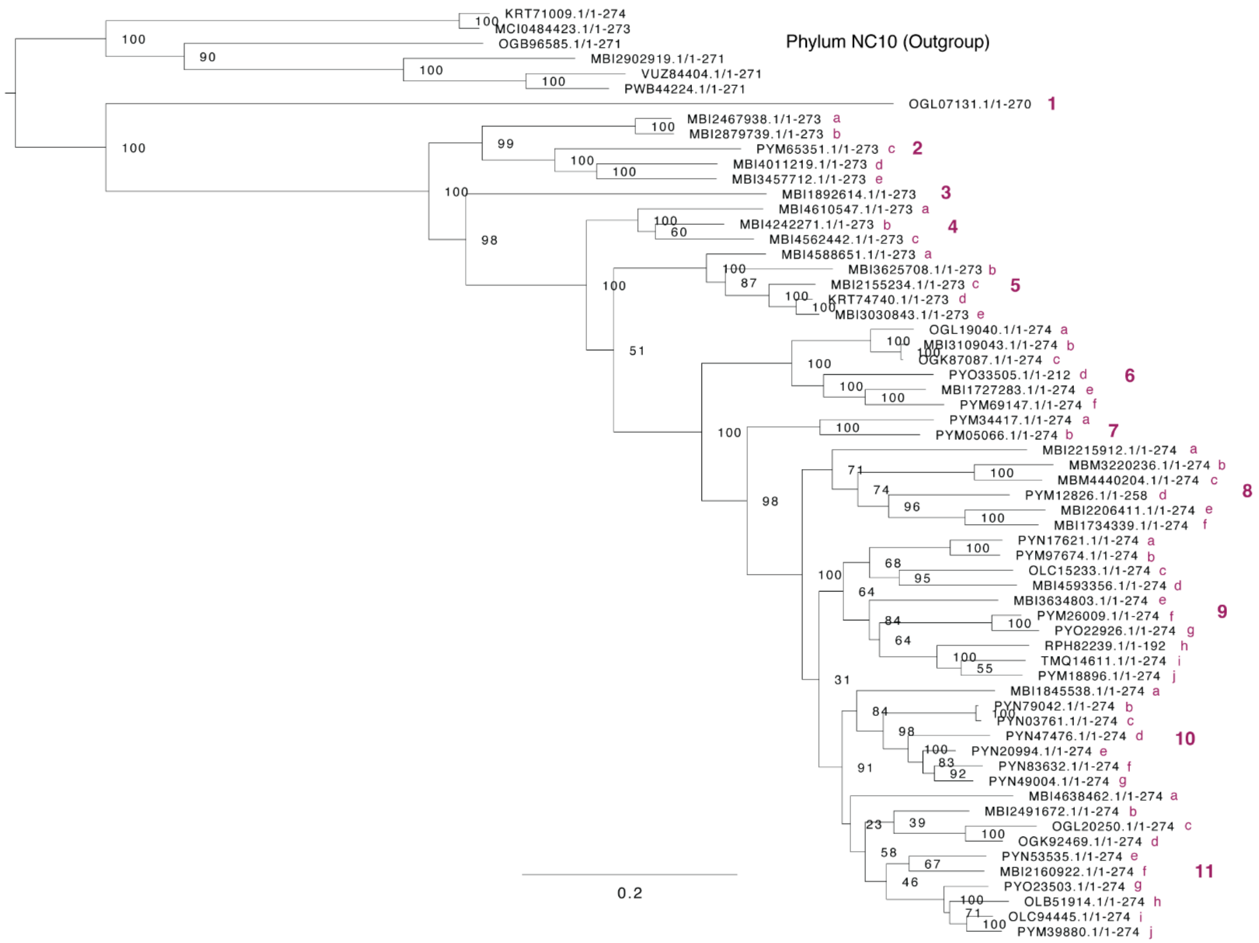

Supplementary Fig. 7. Species tree of 56 rokubacterial species. Rokubacteria contains only uncultured species and thus the species annotation is not available. Instead, individual branches are assigned the accession numbers for the L2 ribosomal protein as the taxon identifier (Supplementary Table 1). Major clusters and branches are additionally assigned labels with numbers and alphabets, which were utilized for HpnP tree reconciliation analyses (Supplementary Figs. 8 and 9). The scale bar represents 0.2 amino acid replacements per site per unit evolutionary time.

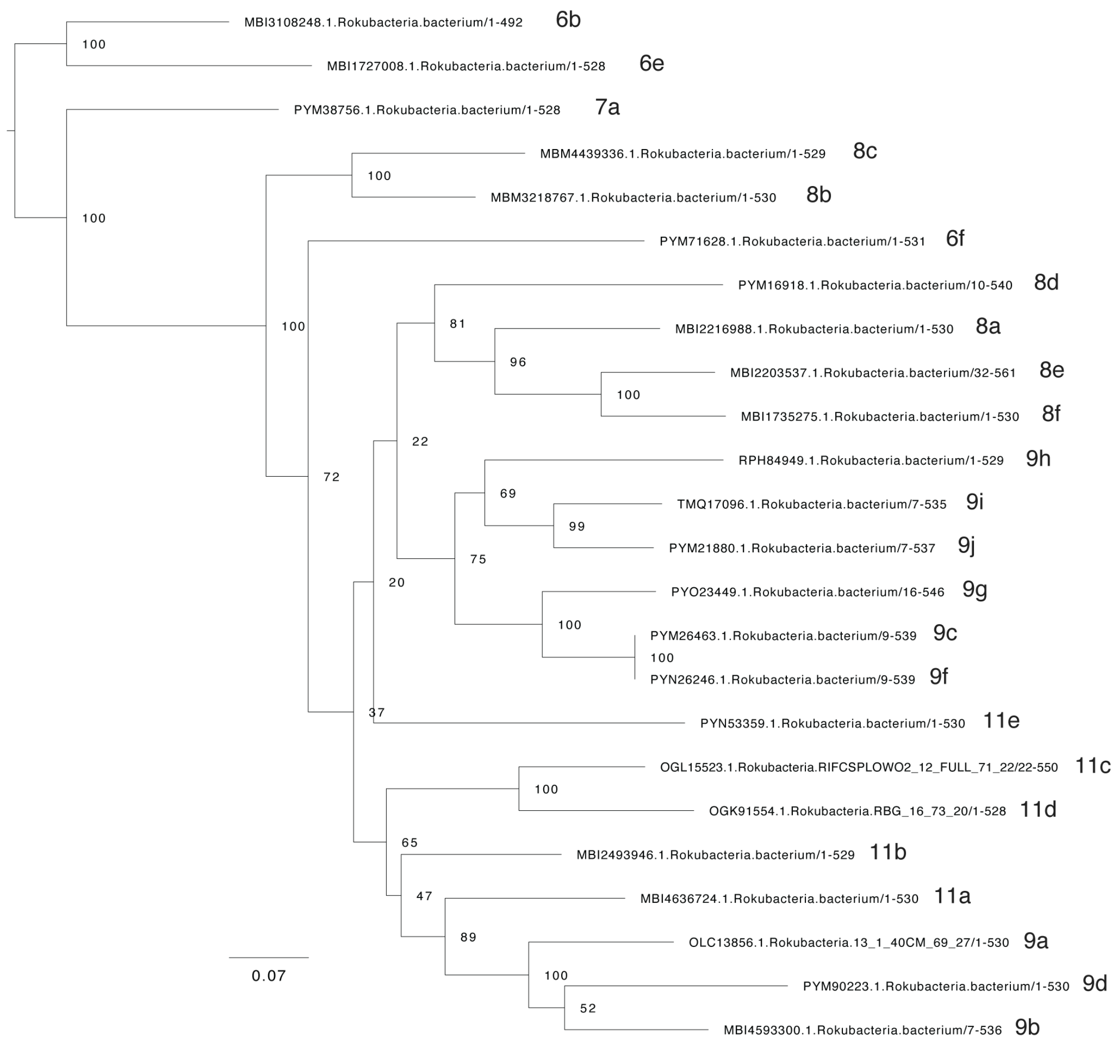

Supplementary Fig. 8. Rokubaterial HpnP tree. The labels next to the species annotation correspond to those in the species tree (Supplementary Fig. 7). The Species annotation includes the HpnP accession number. The tree was rooted to best fit the species tree topology.

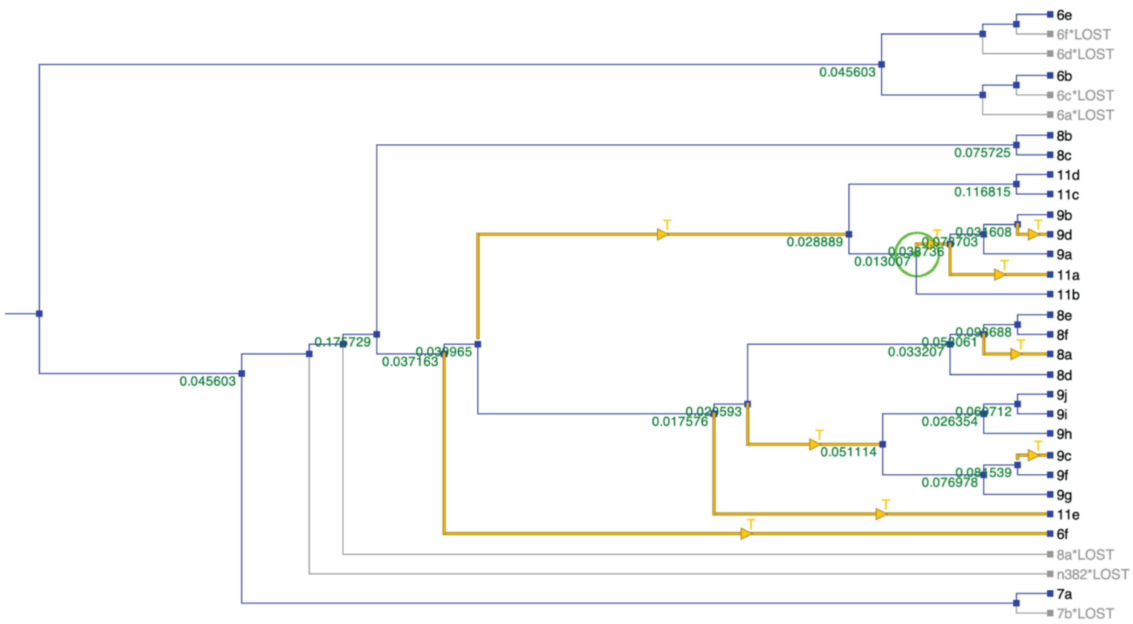

Supplementary Fig. 9. The comparison of rokubacterial HpnP tree and the species tree by Notung. The label T indicates horizontal gene transfer. The labels next to individual branches correspond to those in the species tree (Supplementary Fig. 7).

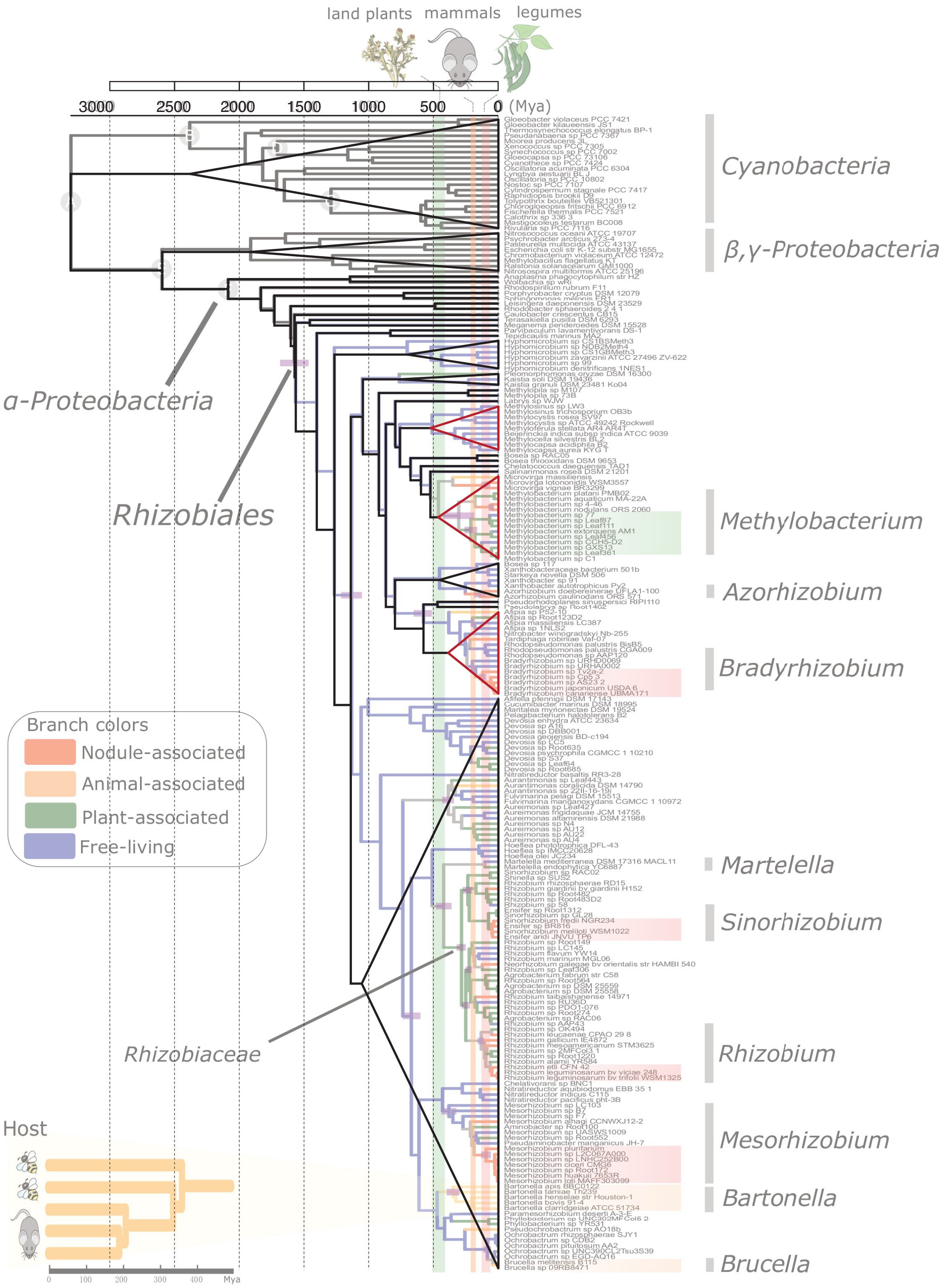

Supplementary Fig. 10. Published dated phylogeny of Cyanobacteria and Alphaproteobacteria[14] and the superimposition of the simplified tree that is used in Fig. 4a. The superimposed three red triangles represent Beijerinckiaceae (top), Methylobacteriaceae (middle) and Nitrobacteraceae

(bottom). In Wang et al., 2020, relaxed molecular clock analyses were performed utilizing this concatenated tree based on the amino acid sequences of 25 universally conserved single-copy genes. Further details are referred to the original study.

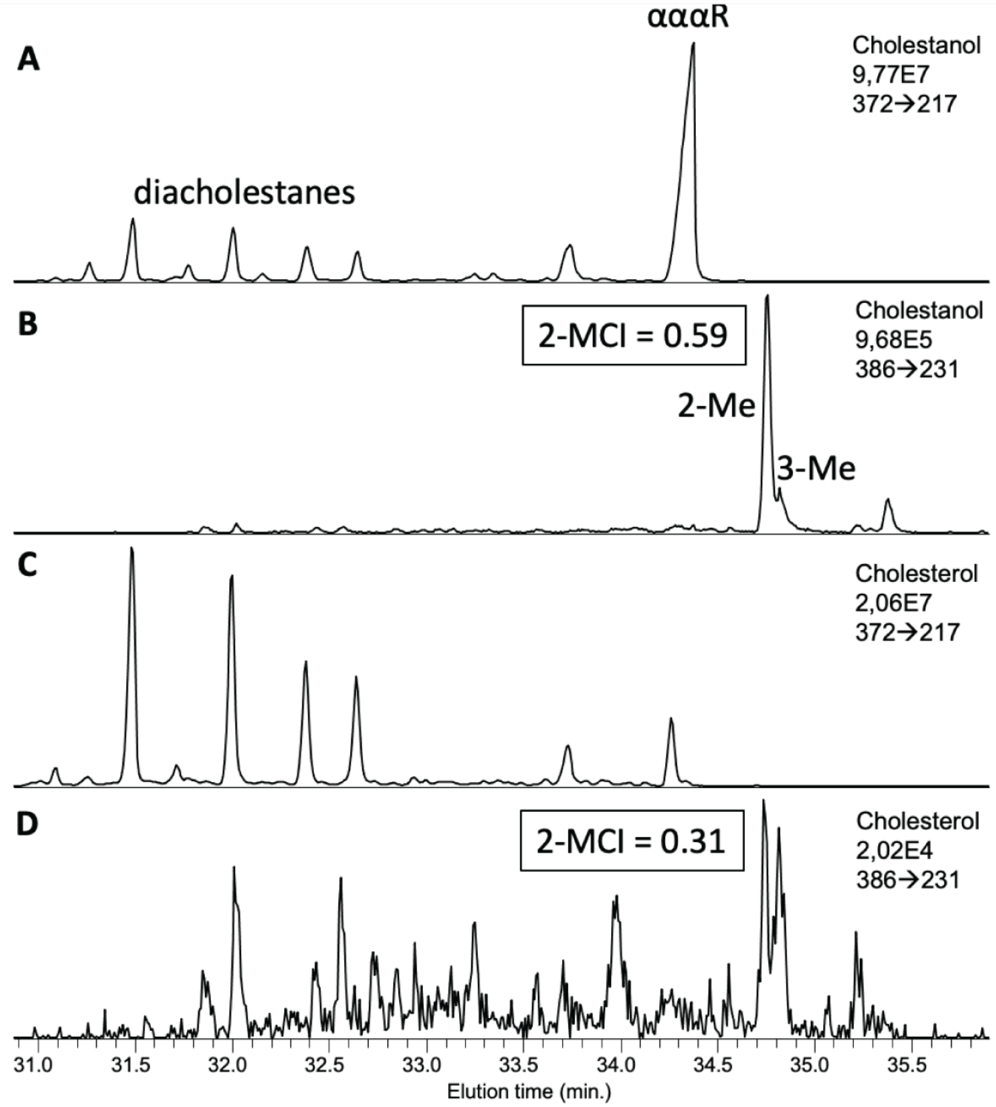

Supplementary Fig 11. Pyrolysis experiment of cholestanol and cholesterol educts. (A) *m/z* 372 → 217 transition showing cholestanes and (B) *m/z* 386 → 231 showing methylcholestanes in cholestanol pyrolysates. (C) *m/z* 372 → 217 transition showing cholestanes and (D) *m/z* 386 → 231 showing methylcholestanes in cholesterol pyrolysates. The 2-methylcholestane index (2-MCI) is calculated analogously to the 2-MHI (%) for the αααR isomers as 2-methylcholestane/(cholestane+2-methylcholestane)*100. Compound abbreviations: αααR, C_{27} 5α(H), 14α(H), 17α(H)-cholestane (20R); 2-Me, 2-methylcholestane; 3-Me, 3-methylcholestane.

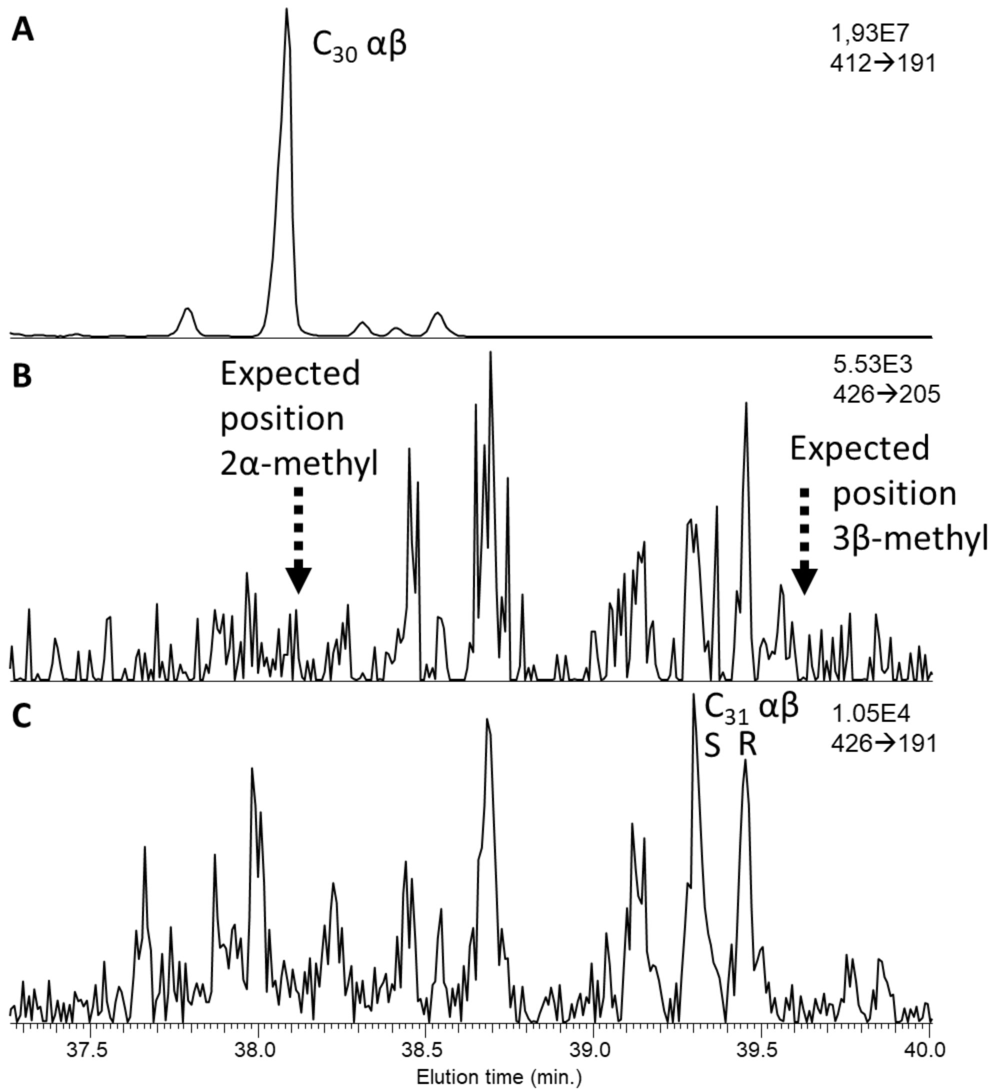

Supplementary Fig. 12. Pyrolysis experiment of diplopterol (1 µL injection from ca. 20 µL total extract, resulting in the overloading of C_{30} αβ-hopane). (A) *m/z* 412 → 191 transition displaying a dominance of the regular C_{30} αβ-hopane isomer. (B) *m/z* 426 → 205 transition displaying the absence of C_{31} methylhopanes with two expected elution times for 2α- and 3β-methylhopanes (according to AGSO reference standard). (C) *m/z* 426 → 191 transition displaying the C_{31} αβ-homohopane R and S isomers. Trace peaks in the *m/z* 426 → 205 transition are likely to mostly represent side-chain methylated hopanoids that yield higher intensities in the *m/z* 426 → 191 transition, although individual compounds are not identified, except for C_{31} αβ-homohopanes.

Supplementary Table captions (see the separate xlsx file for individual tables)

Supplementary Table 1. **SC and HpnP distribution in Rokubacteria**. Accession numbers of SC and HpnP protein sequences for Rokubacteria. This list contains all available genomes in the phylum, regardless of their assembly level because no complete genome is available for this phylum.

Supplementary Table 2. **Taxonomic distribution of SC and HpnP in Hyphomicrobiales in Alphaproteobacteria.** Distribution of SC and HpnP in Hyphomicrobiales in Alphaproteobacteria. The occurrence of SC and HpnP is summarized based on complete genome data and on draft genome data, respectively. The total number of available draft genomes is not counted and instead only the presence/absence of SC or HpnP is suggested in the table.

Supplementary Table 3. **SC and HpnP distribution in Alphaproteobacteria**. Accession numbers of SC and HpnP protein sequences for Alphaproteobacteria. Only families that harbor HpnP-containing species are included in the list. Species that have a complete genome are all included regardless of their containing the SC or HpnP gene. Species that have only a draft genome data are included only if they contain both SC and HpnP genes and also if none of the species that have a complete genome data in the same genus have the HpnP gene. Abbreviation: OSC, oxidosqualene cyclase; CobG, precorrin-3B synthase; CobF, precorrin-6A synthase.

Supplementary Table 4. **Taxonomic distribution of SC and HpnP in Cyanobacteria.** Distribution of SC and HpnP in Cyanobacteria. The occurrence of SC and HpnP is summarized based on complete genome data and on draft genome data, respectively. The total number of available draft genomes is not counted and instead only the presence/absence of SC or HpnP is suggested in the table.

Supplementary Table 5. **SC and HpnP distribution in Cyanobacteria**. Accession numbers of SC and HpnP protein sequences for Cyanobacteria. Species that have a complete genome are all included regardless of their containing the SC or HpnP gene. Species that have only a draft genome data are included only if they contain both SC and HpnP genes and also if none of the species that have a complete genome data in the same genus have the HpnP gene.

Supplementary Table 6. **Total hopane abundance and 2-MHI in the Barney Creek Formation drill core GR7.** Total hopane abundance and the 2-methylhopane index (2-MHI) in the drill core GR7 (Barney Creek Formation). Bold-face indicates samples that have hopanes only in a low abundance but display a high 2-MHI value. Bulk-analyzed samples are not included in our dataset (Fig. 4b). n.d. = not detected.

Supplementary Table 7. **Sample list for the 2-MHI record**. Sample list for the 2-MHI record (Fig. 4b). Samples newly analyzed in this study are shown in brown, while samples from the literature are shown in black. References are shown in Supplementary Information References.
