# Supplementary material for: Genetics re-establish the utility of 2-methylhopanes as cyanobacterial biomarkers before 750 million years ago
Source: Nat Ecol Evol. 2023 Oct 26;7(12):2045–54. doi: 10.1038/s41559-023-02223-5 (PMC10697835; doi:10.1038/s41559-023-02223-5)
Supplement: Supplementary file 2 — Reporting Summary [file 41559_2023_2223_MOESM2_ESM.pdf]

nature portfolio

Corresponding author(s): Yosuke Hoshino

Last updated by author(s): Aug 1, 2023

Reporting Summary

Nature Portfolio wishes to improve the reproducibility of the work that we publish. This form provides structure for consistency and transparency in reporting. For further information on Nature Portfolio policies, see our Editorial Policies and the Editorial Policy Checklist.

Statistics

For all statistical analyses, confirm that the following items are present in the figure legend, table legend, main text, or Methods section.

n/a	Confirmed	
☐	☒	The exact sample size (*n*) for each experimental group/condition, given as a discrete number and unit of measurement
☐	☒	A statement on whether measurements were taken from distinct samples or whether the same sample was measured repeatedly
☐	☒	The statistical test(s) used AND whether they are one- or two-sided<br>*Only common tests should be described solely by name; describe more complex techniques in the Methods section.*
☒	☐	A description of all covariates tested
☐	☒	A description of any assumptions or corrections, such as tests of normality and adjustment for multiple comparisons
☒	☐	A full description of the statistical parameters including central tendency (e.g. means) or other basic estimates (e.g. regression coefficient) AND variation (e.g. standard deviation) or associated estimates of uncertainty (e.g. confidence intervals)
☒	☐	For null hypothesis testing, the test statistic (e.g. *F*, *t*, *r*) with confidence intervals, effect sizes, degrees of freedom and *P* value noted<br>*Give P values as exact values whenever suitable.*
☐	☒	For Bayesian analysis, information on the choice of priors and Markov chain Monte Carlo settings
☒	☐	For hierarchical and complex designs, identification of the appropriate level for tests and full reporting of outcomes
☒	☐	Estimates of effect sizes (e.g. Cohen's *d*, Pearson's *r*), indicating how they were calculated

Our web collection on statistics for biologists contains articles on many of the points above.

Software and code

Policy information about availability of computer code

Data collection	NCBI protein database, NCBI Identical Protein Group database.
Data analysis	Muscle v3.8.31, IQ-TREE v2.1.06, MrBayes v3.2.6, OrthoFinder v2.5.4, FASconCAT-G v1, NOTUNG v2.9.

For manuscripts utilizing custom algorithms or software that are central to the research but not yet described in published literature, software must be made available to editors and reviewers. We strongly encourage code deposition in a community repository (e.g. GitHub). See the Nature Portfolio guidelines for submitting code & software for further information.

Data

Policy information about availability of data

All manuscripts must include a data availability statement. This statement should provide the following information, where applicable:

- Accession codes, unique identifiers, or web links for publicly available datasets
- A description of any restrictions on data availability
- For clinical datasets or third party data, please ensure that the statement adheres to our policy

All data needed to evaluate the conclusions in the paper are present in the paper and/or the Supplementary Materials. Additional and raw data, as well as used sample material, related to this paper may be provided by the authors upon reasonable request.

Human research participants

Policy information about studies involving human research participants and Sex and Gender in Research.

Reporting on sex and gender	n/a
Population characteristics	n/a
Recruitment	n/a
Ethics oversight	n/a

Note that full information on the approval of the study protocol must also be provided in the manuscript.

Field-specific reporting

Please select the one below that is the best fit for your research. If you are not sure, read the appropriate sections before making your selection.

☐ Life sciences ☐ Behavioural & social sciences ☒ Ecological, evolutionary & environmental sciences

For a reference copy of the document with all sections, see nature.com/documents/nr-reporting-summary-flat.pdf

Ecological, evolutionary & environmental sciences study design

All studies must disclose on these points even when the disclosure is negative.

Study description	Trace organic analyses of geological samples
Research sample	Sedimentary rocks from drill cores and outcrops.
Sampling strategy	To obtain a geological trend of 2-methylhopane abundances across the Proterozoic, we exclusively (re)analysed drill core and rock samples already available in the sample collection of the Hallmann and Brocks labs that were known from previous analyses to host indigenous biomarkers.
Data collection	B.J.N., G.V., L.v.M. C.B., and J.J.B. collected and recorded the data.
Timing and spatial scale	We generally tried to include representative samples for each formation by sampling at different stratigraphic heights (depending on formation thickness meters to tens of meters apart) and including different lithologies (e.g. carbonates and shales). Rock samples typically comprise 1 to 3 cm sedimentary thickness and thus typically integrate tens to thousands of years of depositional history depending on local deposition rates.
Data exclusions	Samples that contain anthropogenic contamination are excluded as described in the Supplementary Information.
Reproducibility	Multiple data points are obtained from individual formations.
Randomization	n/a
Blinding	n/a

Did the study involve field work? ☒ Yes ☐ No

Field work, collection and transport

Field conditions	Field conditions were not meaningful for the collection of rock samples.
Location	Multiple locations from Eurasia, Australia, Africa and America. The details are in Supplementary Information.
Access & import/export	Required sample permits were obtained in advance for all outcrop and drill core store sampling campaigns previously conducted by the Brocks and Hallmann groups. Samples were shipped to the laboratory according to local costums regulations.
Disturbance	Most samples where specifically kept in (publically funded) drill core stores for the purpose scientific sampling and analyses. Only

Disturbance	subsamples of core material were taken to minimize disturbance of drill cores. Outcrops samples were obtained by hand (hammer) only with minimal disturbance to the environment.

Reporting for specific materials, systems and methods

We require information from authors about some types of materials, experimental systems and methods used in many studies. Here, indicate whether each material, system or method listed is relevant to your study. If you are not sure if a list item applies to your research, read the appropriate section before selecting a response.

Materials & experimental systems

n/a	Involved in the study
☒	☐ Antibodies
☒	☐ Eukaryotic cell lines
☒	☐ Palaeontology and archaeology
☒	☐ Animals and other organisms
☒	☐ Clinical data
☒	☐ Dual use research of concern

Methods

n/a	Involved in the study
☒	☐ ChIP-seq
☒	☐ Flow cytometry
☒	☐ MRI-based neuroimaging
